# Effect of normal saline and half saline preservation serums on sodium levels, blood pressure, and acidosis in PICU patients

**DOI:** 10.1097/MD.0000000000048090

**Published:** 2026-03-27

**Authors:** Maryam Ghodsi, Effat Hosseinali Beigi, Mohammad Eftekhari, Nasrollah Shafighi, Faezeh Jahanshahi

**Affiliations:** a Department of Pediatrics, School of Medicine, Bahrami Hospital, Tehran University of Medical Sciences, Tehran, Iran; b Prehospital and Hospital Emergency Research Center, School of Medicine, Imam Khomeini Hospital Complex, Tehran University of Medical Science, Tehran, Iran; c Department of Internal Medicine, Shahid Beheshti University of Medical Sciences, Tehran, Iran.

**Keywords:** blood pressure, half saline, normal saline, pediatric Intensive Care Unit (PICU), sodium levels

## Abstract

**Background:**

This study aimed to evaluate the differential effects of normal saline (N/S) (0.9% sodium chloride) and half saline (H/S) (0.45% sodium chloride) preservation solutions on sodium levels, blood pressure, and acid-base balance in critically ill pediatric patients.

**Methods:**

A double-blind interventional clinical trial involving 88 pediatric patients was conducted in the pediatric intensive care unit. Patients were randomly assigned to receive either N/S (Group A) or H/S (Group B). Sodium, potassium, chloride, bicarbonate, pH, and blood pressure were measured at baseline and 6, 24, 48, and 72 hours. The primary outcome was the change in serum sodium concentration, and secondary outcomes included blood pressure and acid-base status.

**Results:**

At 72 hours, sodium levels were significantly higher in the N/S group (137.63 ± 2.99 mEq/L) compared to the H/S group (135.33 ± 2.46 mEq/L; *P* = .007). Blood pressure was significantly higher in the N/S group at 24 hours (98.78 ± 8.08 mm Hg vs 92.70 ± 9.24 mm Hg; *P* = .002). No significant differences were observed in potassium levels, pH, or bicarbonate concentrations. The incidence of hyponatremia at 72 hours was similar in both the groups (15.9% for N/S vs 13.6% for H/S).

**Conclusion:**

Normal saline administration resulted in higher sodium levels and increased systolic blood pressure than H/S administration. These findings underscore the importance of tailored fluid therapy in critically ill pediatric patients, highlighting the potential effects of fluid choice on sodium balance and hemodynamics.

## 1. Introduction

Effective management of critically ill pediatric patients requires precise monitoring and regulation of physiological parameters such as electrolyte balance, blood pressure, and acid-base homeostasis.^[1]^ In Pediatric Intensive Care Units (PICUs), the administration of intravenous fluids is the cornerstone of therapeutic management, as it helps to stabilize the patient condition and prevent or correct fluid and electrolyte imbalances. The choice of intravenous fluids, particularly preservation solutions, can significantly affect the regulation of sodium levels, blood pressure, and acid-base status in critically ill children.^[2]^ Given the high vulnerability of this population to rapid changes in these parameters, understanding the effects of various preservation fluids is crucial for optimizing treatment protocols and improving patient outcomes.^[3]^

Sodium, a key electrolyte, plays a fundamental role in maintaining osmotic balance, regulating blood pressure, and supporting proper neural and muscular function.^[4]^ In critically ill pediatric patients, disturbances in sodium homeostasis, whether in the form of hyponatremia (low sodium) or hypernatremia (high sodium), can have severe consequences.^[5]^ These include cerebral edema, seizures, cardiovascular instability, and renal dysfunction.^[6]^ Pediatric patients, particularly those in the PICU, are particularly susceptible to such imbalances owing to their smaller body size, immature renal function, and higher metabolic rates. These children are at increased risk of complications from electrolyte disturbances because of their altered fluid distribution and the impact of underlying critical illnesses.^[7,8]^

Two commonly used intravenous fluids in pediatric care are normal saline (N/S) (0.9% sodium chloride) and half saline (H/S) (0.45% sodium chloride). While both solutions are isotonic, their sodium concentrations differ, which may influence their effects on fluid balance and electrolyte regulation. N/S with a higher sodium content is often used for rapid fluid resuscitation or to address sodium deficits.^[9,10]^ H/S, with a lower sodium concentration, is used when a more controlled approach to sodium intake is necessary, particularly in cases where there is concern about sodium overload or hypernatremia.^[11]^ However, the differential effects of these 2 fluids on sodium levels, blood pressure, and acid–base status in pediatric patients remain underexplored, particularly within the critical care context of the PICU.^[12]^

In adult populations, studies have assessed the effects of N/S and other intravenous fluids on various clinical outcomes including sodium imbalances, fluid overload, and acid–base disturbances. However, the pediatric population is distinct from adults in several ways. Children, especially those in the PICU, have unique physiological characteristics, such as a higher surface-area-to-volume ratio, which influences their response to fluid administration.^[13]^ Additionally, children kidneys are less mature than those of adults, which may result in a reduced ability to excrete excess sodium or regulate fluid balance efficiently. Given these differences, it is essential to focus specifically on the pediatric population, as fluid and electrolyte disturbances in critically ill children can lead to rapid deterioration, organ failure, and even death if not managed appropriately.^[14,15]^

The primary aim of this study was to investigate the differential effects of N/S and H/S preservation solutions on sodium levels, blood pressure, and acidosis in pediatric patients admitted to the PICU at Bahrami Children’s Hospital, Tehran. By examining these effects, this study seeks to provide much-needed evidence on the optimal intravenous fluid strategy for critically ill children. This study contributes to the growing body of literature on pediatric fluid management, particularly in intensive care settings, where the need for tailored therapeutic approaches is paramount. Understanding how sodium levels, blood pressure, and acid–base balance are influenced by different preservation solutions in children can guide clinical decision-making and improve therapeutic outcomes in critically ill pediatric patients.

## 2. Methods

This study was designed as a double-blind interventional clinical trial conducted in the PICU of Bahrami Children’s Hospital in Tehran in 2023. This study focused on evaluating the effects of N/S and H/S preservation serum on sodium levels and other clinical outcomes in critically ill pediatric patients.

### 2.1. Study participants

The inclusion criteria for the study were:

- Age between 3 months and 18 years.
- Requirement for intravenous fluid administration.

Exclusion criteria included:

- Serum sodium levels below 130 mmol/L or above 150 mmol/L.
- Diagnosis of diabetes insipidus or diabetic ketoacidosis.
- Renal disease requiring dialysis.
- Disorders with excessive urinary sodium secretion (e.g., Addison disease, congenital adrenal hyperplasia, and Bartter syndrome).
- Patients undergoing or having undergone neurosurgery.
- Chemotherapy patients with hydration needs.
- Severe liver disease.
- Patients expected to receive maintenance fluids for <6 hours.

### 2.2. Trial design

The study was approved by the local ethics review board of Tehran University of Medical Sciences in Iran (IR.TUMS.CHMC.REC.1400.145). This study followed the ethical standards set by the Declaration of Helsinki and Good Clinical Practice. The study was registered with the Iranian Registry of Clinical Trials (www.irct.ir) under the identifier IRCT20230414057904N1.

### 2.3. Randomization and group allocation

A total of 88 pediatric patients meeting the inclusion criteria were recruited for this study. These patients were randomly assigned to 1 of 2 groups, N/S (Group A) or H/S (Group B), using a block randomization method with varying block sizes. Randomization was performed using the RANDBLOCK tool within Randomizer software to ensure unbiased allocation. Patients were stratified into 2 groups based on their general condition (good or bad, as per the Pediatric Risk of Mortality 3 score). This stratification was conducted separately to ensure a balanced representation of both conditions across the groups.

The treatment physician, who was blinded to the group assignment, managed the allocation of the intravenous fluids. Fluid administration was continued for up to 72 hours or until the patient received at least 50% of the prescribed saline solution.

### 2.4. Fluid administration and monitoring

The prescribed intravenous fluids, N/S (0.9% sodium chloride) and H/S (0.45% sodium chloride), were administered based on clinical decisions. Sodium, potassium, chloride, bicarbonate (HCO_3_), pH, and blood pressure levels were measured at baseline and then at 6, 24, 48, and 72-hours posttreatment. This data collection aimed to evaluate the changes in electrolyte concentrations and hemodynamic parameters over time.

In addition to these measurements, patients were regularly assessed for signs of dehydration or overhydration. Information was recorded in patient charts following standard clinical routines.

### 2.5. Primary and secondary outcomes

The primary outcome of the study was the change in serum sodium concentration and occurrence of hyponatremia (serum sodium < 130 mmol/L) over 72 hours or upon receiving at least 50% of the intended fluid dose. The secondary outcomes included changes in blood pressure, HCO_3_ concentration, pH, and chloride concentration.

### 2.6. Sample size calculation

The sample size was calculated based on a similar study and the expected mean difference in serum sodium concentration between the N/S and H/S groups (effect size 1.1). A significance level of 0.05 and power of 0.80 were considered. The required sample size per group was calculated to be 52, with a 15% dropout rate, resulting in a total sample size of 60 per group for a total of 88 patients.

### 2.7. Data analysis

Data were analyzed using the Statistical Package for Social Sciences (SPSS) version 14. The normality of the data distribution was tested using the Kolmogorov–Smirnov test. Quantitative variables were described using means and standard deviations or medians and interquartile ranges, whereas qualitative variables were summarized by frequency and percentage.

Within-group changes were analyzed using the Friedman test, and between-group differences and trends were assessed using repeated-measures analysis of variance. To evaluate the impact of the type of fluid administered after adjusting for potential confounders (age, sex, underlying conditions, Pediatric Risk of Mortality score), a generalized estimating equation model was applied. Statistical significance was set at *P* < .05.

### 2.8. Ethical considerations

This study adhered to ethical standards, ensuring that all patient data were kept confidential throughout the research process. Only the aggregate results have been reported in the literature. No personal identifiers were collected from the patient questionnaires. All procedures were performed in accordance with the Declaration of Helsinki and informed consent was obtained from the guardians of all participating patients. Children and parents were assured that the information obtained would remain confidential from the project executives. They were informed about the research objectives and methodology, and subsequently written consent letters were obtained from them.

## 3. Results

### 3.1. Demographic characteristics

**Figure 1.**
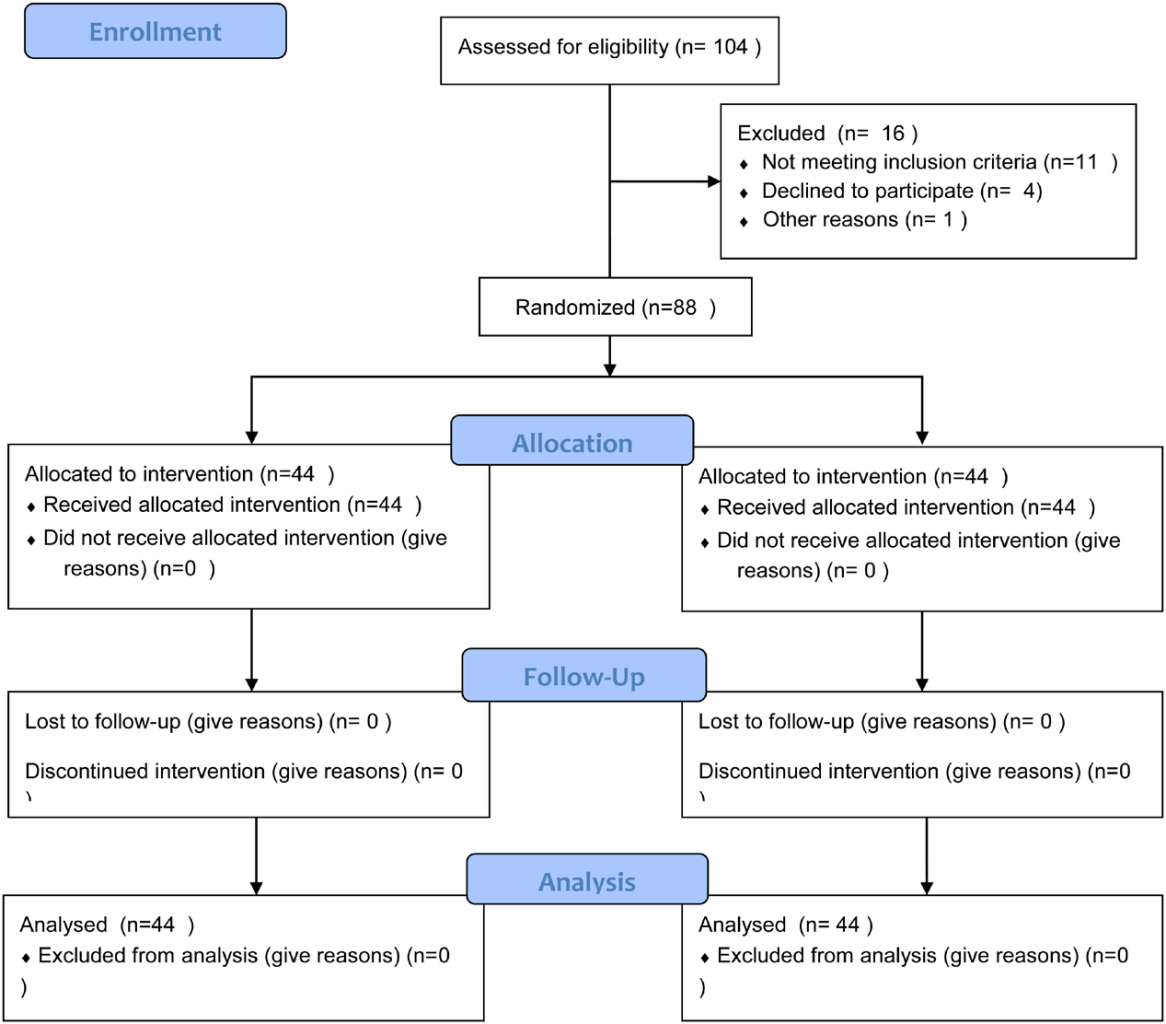
CONSORT flow diagram.

A total of 88 patients were included in the study, of which 57.1% (50 patients) were boys and 42.9% (38 patients) were girls (Fig. 1). The median age distribution revealed that 40.5% of the patients were under 1 year old, 35.7% were between 1 and 5 years old, 17.9% were between 5 and 10 years old, and 6% were over 10 years old. The most common age group was infants under 1 year of age.

The average weight of the patients was 10.7 ± 10 kg in the H/S group and 10.4 ± 12.2 kg in the N/S group. The mean height was 81.5 ± 35 cm for the H/S group and 81.5 ± 41 cm for the N/S group. There were no statistically significant differences in sex, weight, or height between the 2 groups (*P* > .05) (Table 1).

**Table 1:** Baseline characteristics of patients participated in this study.

Variables		D5 + H/S	D5 + N/S	*P* value
Sex	Male	23 (57.5%)	25 (56.8%)	.95
	Female	17 (42.5%)	19 (43.2%)	
Weight (kg)		10.7 (10)	10.4 (12.2)	.42
Height (cm)		81.5 (35)	80.6 (41)	.75

H/S = half saline, N/S = normal saline.

### 3.2. Admission diagnoses

The patients were admitted to the PICU for a variety of critical conditions requiring intravenous fluid therapy. The primary reasons for admission are summarized in Table 2 below, stratified by group. There were no significant differences in admission diagnoses between the N/S and H/S groups (*P* > .05 for all categories), ensuring comparability. The most common diagnoses were infections (e.g., pneumonia, sepsis) and dehydration, reflecting typical PICU presentations in this population.

**Table 2:** Admission diagnoses by group.

Diagnosis category	N/S group (n = 44)	H/S group (n = 44)	Total (n = 88)	*P* value
Infection (e.g., pneumonia, sepsis)	18 (40.9%)	20 (45.5%)	38 (43.2%)	.68
Dehydration	12 (27.3%)	10 (22.7%)	22 (25.0%)	.63
Diarrhea/gastroenteritis	6 (13.6%)	5 (11.4%)	11 (12.5%)	.75
Trauma (e.g., head injury, fractures)	4 (9.1%)	5 (11.4%)	9 (10.2%)	.73
Respiratory distress (e.g., asthma exacerbation)	3 (6.8%)	3 (6.8%)	6 (6.8%)	1.00
Other (e.g., metabolic disorders, postsurgical)	1 (2.3%)	1 (2.3%)	2 (2.3%)	1.00

Note: Percentages are within-group. *P* values from chi-square tests comparing groups.

H/S = half saline, N/S = normal saline.

### 3.3. Sodium levels

**Figure 2.**
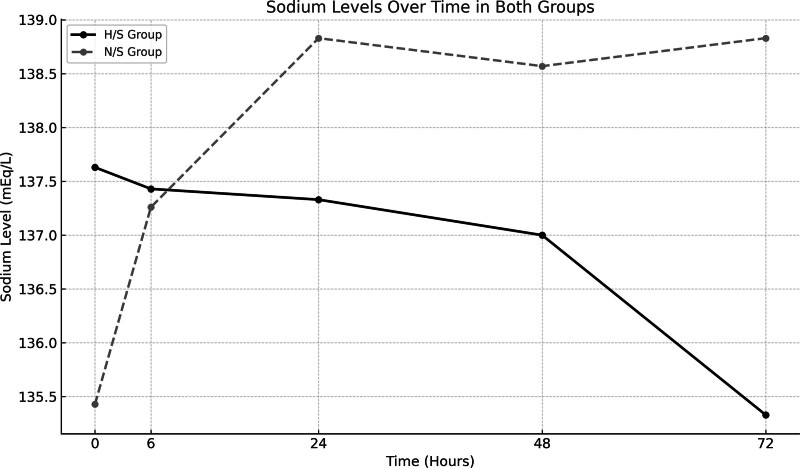
Sodium levels over time in normal saline and half saline preservation serum groups.

Sodium levels were measured at baseline and 6, 24, 48, and 72-hours posttreatment. A significant difference in sodium levels was observed at 72 hours between the 2 groups (*P* = .007). Sodium concentration in the H/S group was significantly lower than that in the N/S group at this time point. The changes in sodium levels from baseline to 72 hours were significant for both groups, with the N/S group showing a higher sodium concentration at 72 hours (137.63 ± 2.99 mEq/L vs 135.33 ± 2.46 mEq/L in the H/S group). However, no significant differences were found at other time points (6, 24, and 48 hours) (Fig. 2).

### 3.4. Potassium levels

Potassium levels were assessed at the same time intervals. No significant differences were observed between the 2 groups in terms of *K* concentration throughout the study period (*P* > .05). Both groups maintained similar potassium levels at 72 hours: 4.17 ± 0.52 mEq/L in the H/S group and 4.01 ± 0.63 mEq/L in the N/S group (Table 3).

**Table 3:** Potassium concentration in different time before and after the intervention.

*K* (h)	D5 + N/S(M ± SD)	D5 + H/S(M ± SD)	*P* value
0	4.01 ± .63	4.17 ± .52	.206
6	3.73 ± .59	4.00 ± .52	.165
24	3.93 ± .71	4.09 ± .50	.301
48	3.93 ± .60	4.11 ± .46	.247
72	4.05 ± .50	4.27 ± .36	.163

H/S = half saline, N/S = normal saline, SD = standard deviation.

### 3.5. Acid–Base Status

Acid–base balance, indicated by pH and HCO_3_, was evaluated over time. No significant differences were observed in the pH or HCO_3_ levels between the 2 groups at any time point (*P* > .05, for all comparisons). The mean pH values were 7.30 ± 0.16 for the H/S group and 7.31 ± 0.10 for the N/S group at baseline, with minimal variation at later time points (Table 4).

**Table 4:** pH and HCO_3_ levels over time in both groups.

Time (h)	D5 + N/S(M ± SD)	D5 + H/S(M ± SD)	*P* value	D5 + N/S(M ± SD)	D5 + H/S(M ± SD)	*P* value
0	7.31 ± 0.10	7.30 ± 0.15	.877	17.84 ± 4.90	19.04 ± 4.31	.275
6	7.35 ± 0.05	7.33 ± 0.09	.480	18.77 ± 6.01	20.45 ± 4.97	.311
24	7.37 ± 0.07	7.35 ± 0.05	.185	19.05 ± 3.30	19.97 ± 5.01	.416
48	7.36 ± 0.09	7.39 ± 0.08	.223	18.98 ± 3.93	19.74 ± 4.61	.548
72	7.37 ± 0.11	7.39 ± 0.06	.646	19.86 ± 4.49	18.99 ± 3.27	.556

H/S = half saline, N/S = normal saline, SD = standard deviation.

### 3.6. Blood pressure

**Figure 3.**
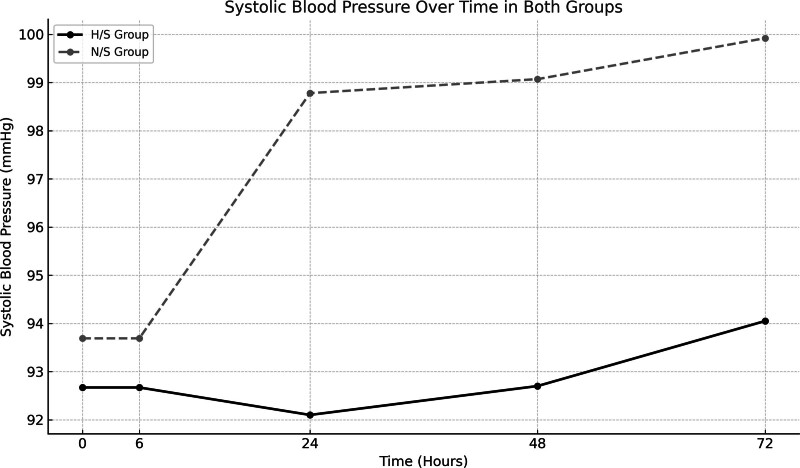
Systolic blood pressure over time in normal saline and half saline preservation serum groups.

Blood pressure was measured at baseline and at 6, 24, 48, and 72 hours. A significant difference was found in systolic blood pressure at 24 hours, where the N/S group exhibited higher systolic blood pressure compared to the H/S group (98.78 ± 8.08 mm Hg vs 92.70 ± 9.24 mm Hg; *P* = .002). Systolic blood pressure in the N/S group remained higher at other time points, although the differences were not statistically significant (Fig. 3).

In terms of hypertension classification, 36.2% of patients in the N/S group had elevated blood pressure, with 18.1% having stage 1 hypertension and 2.2% having stage 2 hypertension. In contrast, only 20.3% of the patients in the H/S group had elevated blood pressure, with 18.1% in the prehypertension stage and 2.2% in stage 1 hypertension. The frequency of stage 2 hypertension was significantly higher in the N/S group (*P* < .0001).

### 3.7. Hyponatremia incidence

Hyponatremia, defined as serum sodium level < 135 mEq/L, was monitored throughout the study. At 72 hours, the incidence of hyponatremia was 15.9% in the N/S group and 13.6% in the H/S group, with no significant difference between the groups (*P* = .08). These findings suggest that the administration of H/S does not present additional risks of hyponatremia compared with N/S.

## 4. Discussion

This study aimed to evaluate the effects of N/S and H/S preservation solutions on sodium levels, blood pressure, and acid-base balance in critically ill pediatric patients in the PICU. These findings provide important evidence regarding the optimal use of these fluids in managing electrolyte and hemodynamic parameters in this vulnerable population.

The results of the study indicate significant differences in sodium regulation and blood pressure between the 2 groups, with the N/S group showing higher sodium levels and elevated systolic blood pressure than the H/S group. However, no significant differences were observed in potassium, pH, or HCO_3_ levels between the 2 groups. These findings are particularly relevant for clinicians managing fluid therapy in critically ill pediatric patients, where electrolyte balance and blood pressure control are crucial to patient outcomes.

The findings of this study contribute to a growing body of research on the choice of intravenous fluids in critically ill pediatric patients. In particular, the observed higher sodium levels and elevated blood pressure in the N/S group were consistent with findings from multiple studies, such as those by McNab et al^[16]^ and Kumar et al,^[17]^ who also observed that N/S is associated with higher sodium concentrations, which may be linked to increased blood pressure.

Moritz et al demonstrated that administration of hypotonic solutions could lead to severe neurological complications, including hyponatremia, particularly in hospitalized children. This highlights the importance of choosing an appropriate intravenous fluid, especially in pediatric patients with immature renal function and high fluid turnover.^[18,19]^

McNab et al conducted a large-scale randomized clinical trial and found that the administration of N/S was associated with a lower risk of hyponatremia than H/S in children, which supports our finding of no significant difference in the incidence of hyponatremia between the 2 groups. However, our study extends this knowledge by demonstrating that despite similar rates of hyponatremia, the N/S group experienced higher systolic blood pressure, which is a concern given the potential for fluid overload in critically ill patients.^[16]^ In addition, Lehtiranta et al conducted a pragmatic clinical trial involving 614 children in Finland comparing isotonic and hypotonic fluids. They found that while the incidence of electrolyte disturbances was lower in the hypotonic fluid group, clinical complications related to hypernatremia and hypokalemia were significantly more frequent in the isotonic fluid group.^[20]^ This finding, while focusing on a different population, is consistent with the current study observation that N/S, with its higher sodium content, can contribute to sodium retention and a greater risk of hypernatremia, particularly in critically ill patients.

In contrast, studies by Kumar et al^[17]^ and Langer et al^[21]^ found similar findings where the use of N/S led to higher sodium levels, but the long-term impact on blood pressure was not as consistently noted. This suggests that while N/S may effectively increase sodium concentrations in the short term, its long-term effects on fluid balance and blood pressure regulation may vary based on the patient underlying conditions.

The differential effects of N/S and H/S on sodium levels and blood pressure can be explained by the differences in their sodium content. Sodium is a key determinant of extracellular fluid volume, osmolarity, and vascular tone. The higher sodium concentration in N/S likely promotes greater retention of water in the extracellular space, leading to increased blood volume and subsequently, higher blood pressure, particularly in critically ill patients.^[22]^ This is consistent with the work of Alobaidi et al,^[23]^ who showed that fluid resuscitation with higher sodium content tends to increase blood pressure, which could be beneficial in cases of shock, but may also increase the risk of hypertension.

In contrast, H/S, with its lower sodium concentration, provides a more controlled release of sodium, which may mitigate the risk of fluid overload and hypernatremia. The lack of significant differences in potassium levels and acid–base balance between the groups suggests that these parameters are not as sensitive to changes in sodium intake, and that both N/S and H/S appear to be equally effective in maintaining these values within the normal range.^[24]^

While the observed differences in sodium levels (mean difference of 2.3 mEq/L at 72 hours) and systolic blood pressure (mean difference of 6.08 mm Hg at 24 hours) were statistically significant, their clinical relevance may be limited in certain contexts, as these changes fall within ranges that might not always necessitate immediate intervention in stable patients. However, in critically ill pediatric patients, even small shifts in these parameters can accumulate over time and contribute to complications such as fluid overload or subtle hemodynamic instability, particularly in those with immature renal function or comorbidities. This underscores the need for interpreting statistical significance alongside clinical judgment and highlights the value of our findings in guiding nuanced fluid selection to prevent potential adverse outcomes.

The clinical implications of these findings are significant in the management of critically ill pediatric patients in PICUs. Given the observed differences in sodium regulation and blood pressure, it is essential to tailor intravenous fluid therapy according to the individual needs of the patient. For patients at risk of hypernatremia or those requiring more controlled sodium intake, H/S may be a better choice as it offers a more gradual increase in sodium levels without significantly affecting blood pressure. However, N/S may be more appropriate for patients requiring rapid fluid resuscitation or those with sodium deficits that need to be addressed quickly.^[25]^

The higher incidence of elevated systolic blood pressure in the N/S group highlights the need for careful monitoring of blood pressure in these patients, particularly in those with underlying cardiovascular risk factors. The results suggest that while N/S is effective in correcting sodium imbalances, it may also require additional interventions to manage blood pressure, especially in pediatric patients with fluid overload or preexisting hypertension.

Despite the strengths of this study, it has several limitations must be acknowledged. This study was conducted in a single PICU, which may limit the generalizability of the results to other settings or populations. Additionally, the relatively small sample size (n = 88) may have limited the statistical power to detect subtler differences between groups, although it was calculated to achieve adequate power for the primary outcome. Furthermore, the study duration was limited to 72 hours, and extending the observation period to 5 days or longer could potentially reveal more pronounced clinical differences due to cumulative effects on electrolyte balance and hemodynamics. Future studies with longer follow-up periods and larger sample sizes are needed to better understand the long-term effects of intravenous fluids.

Furthermore, the study did not consider the potential impact of underlying comorbidities or concurrent medications, which could have influenced the observed effects. Future research should control for these variables and explore the effects of N/S and H/S in more diverse patient populations.

Future research should focus on multicenter, randomized controlled trials with larger sample sizes to confirm the findings of this study and explore the broader applicability of these results. Additionally, long-term studies with extended durations, such as 5 days or more, evaluating the effects of N/S and H/S on renal function, cardiovascular outcomes, and overall survival would provide more comprehensive data on the safety and efficacy of these fluids in critically ill children, potentially uncovering more significant clinical differences.

Moreover, studies examining the use of balanced crystalloids as an alternative to N/S and H/S in pediatric patients would offer valuable insights into optimal fluid management strategies in the PICU. Investigating the role of these fluids in different clinical conditions, such as renal dysfunction or shock, would further help tailor fluid therapy for critically ill pediatric patients.

## 5. Conclusion

In conclusion, this study provided crucial evidence regarding the differential effects of N/S and H/S preservation solutions on sodium regulation, blood pressure, and acidosis in critically ill pediatric patients. These findings suggest that while N/S may be associated with higher sodium levels and increased blood pressure, H/S offers a more controlled approach to sodium administration with similar outcomes in other clinical parameters. These results contribute to ongoing efforts to optimize intravenous fluid therapy in pediatric intensive care, emphasizing the need for individualized treatment plans to minimize risks and improve patient outcomes.

## Acknowledgments

We would like to extend our sincere gratitude to all participants of this study.

## Author contributions

**Conceptualization:** Maryam Ghodsi, Effat Hosseinali Beigi, Faezeh Jahanshahi.

**Data curation:** Effat Hosseinali Beigi, Nasrollah Shafighi.

**Funding acquisition:** Nasrollah Shafighi.

**Investigation:** Maryam Ghodsi, Effat Hosseinali Beigi, Mohammad Eftekhari, Faezeh Jahanshahi.

**Methodology:** Effat Hosseinali Beigi, Mohammad Eftekhari, Faezeh Jahanshahi.

**Project administration:** Mohammad Eftekhari.

**Writing – original draft:** Effat Hosseinali Beigi, Mohammad Eftekhari, Nasrollah Shafighi, Faezeh Jahanshahi.

**Writing – review & editing:** Mohammad Eftekhari, Nasrollah Shafighi.
